# Laparoscopic Intra-Mediastinum Omental Filling Repair for Spontaneous Esophageal Rupture: A Case Report

**DOI:** 10.70352/scrj.cr.24-0131

**Published:** 2025-05-01

**Authors:** Demba Ishimine, Hiroki Sunagawa, Maina Teruya, Keigo Hayashi

**Affiliations:** Department of Digestive and General Surgery, Nakagami Hospital

**Keywords:** spontaneous esophageal rupture, laparoscopy, omental filling

## Abstract

**INTRODUCTION:**

Spontaneous esophageal rupture is a rare but life-threatening condition with a high mortality rate. While conservative and endoscopic therapies have been reported, surgical treatment remains essential. The optimal approach involves esophageal defect repair and mediastinal drainage, which is performed via laparotomy, thoracotomy, laparoscopy, or a combination of these techniques. We report a case of laparoscopic intramediastinal omental filling for a spontaneous esophageal rupture that was challenging to close.

**CASE PRESENTATION:**

A 62-year-old man presented with sudden-onset right-sided abdominal pain. Computed tomography and esophagography revealed a spontaneous rupture of the right wall of the lower esophagus. Laparoscopic surgery was performed 4 h following symptom onset. A partially necrotic area was identified in the lower esophagus. Given the difficulty of suturing the necrotic esophageal wall, the mediastinum was filled with the greater omentum to cover the perforation site. The patient had no significant postoperative complications and was discharged on the 24th postoperative day.

**CONCLUSIONS:**

Laparoscopic omental filling repair is a viable option for esophageal rupture when primary suture closure is not feasible.

## INTRODUCTION

Spontaneous esophageal rupture is a relatively rare but severe condition involving full-thickness esophageal wall perforation due to a sudden rise in intraluminal pressure. Surgical intervention is the standard treatment, with recent reports highlighting thoracoscopic surgery, while laparoscopic approaches remain uncommon. This report presents a case of successful laparoscopic intramediastinal omental filling for a spontaneous esophageal rupture, where primary closure was not feasible, thus resulting in a favorable outcome. We also explore the indications and surgical approaches for spontaneous esophageal rupture.

## CASE PRESENTATION

A 62-year-old man with no significant medical history presented with a sudden-onset right-sided abdominal pain. He was transferred to our emergency unit 1 h later. His vital signs were: body temperature 36.7°C, blood pressure 113/78 mmHg, pulse 128/min, and SpO2 95% on room air. Physical examination revealed generalized abdominal tenderness, muscular defense, and a snow-grasping sensation in the neck. Blood tests revealed a white blood cell count of 21,130/mL, a pH of 6.81, and a lactate level of >20 mmol/L.

**Fig. 1:**
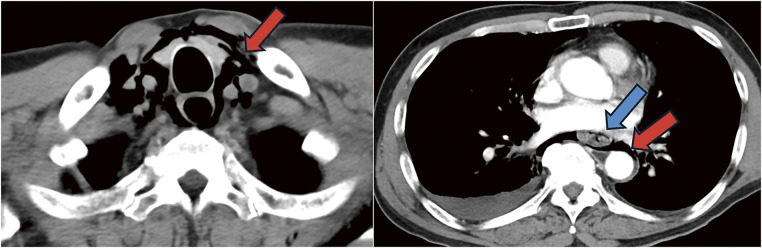
Computed tomography (CT) scan. CT revealed pneumomediastinum from the pharynx to the esophagogastric junction (the red arrow). The blue arrow shows the thickness and the disruption of the esophageal wall.

**Fig. 2:**
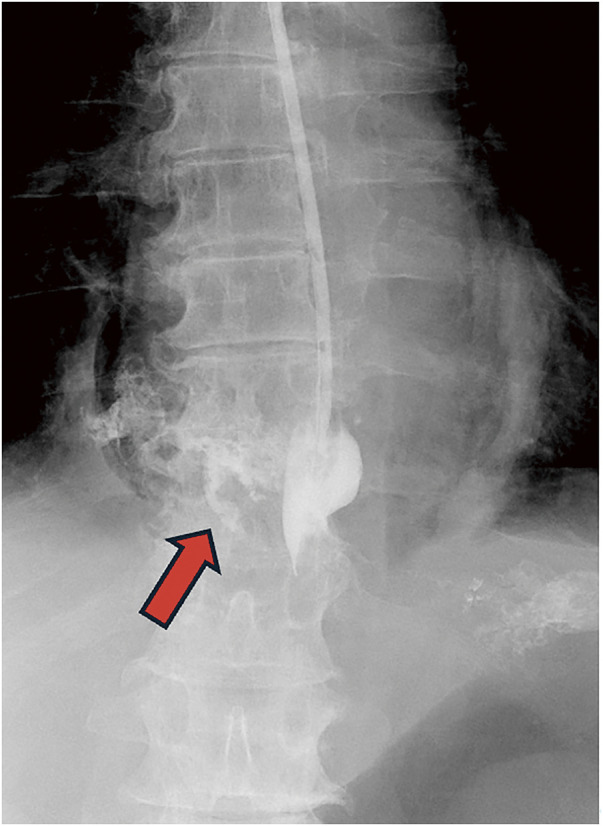
Contrast study. The red arrow shows the extravasation of the contrast from the esophagus to the mediastinum.

Contrast-enhanced computed tomography (CT) revealed emphysema extending from the pharynx through the neck and mediastinum, thickening and disruption of the lower esophageal wall, fluid accumulation in the right thoracic cavity, and pneumothorax (**Fig. 1**). A gastric tube was inserted into the esophagus for decompression and drainage. In cases of minor leaks, the tube was left in place for conservative management. Continuous contrast injection via the gastric tube revealed leakage from the right side of the lower esophagus into the mediastinum (**Fig. 2**), thus confirming spontaneous esophageal rupture. Consequently, an emergency surgery was performed. As the perforation was suspected to be on the right side of the lower esophagus, laparoscopic surgery was chosen.

**Fig. 3:**
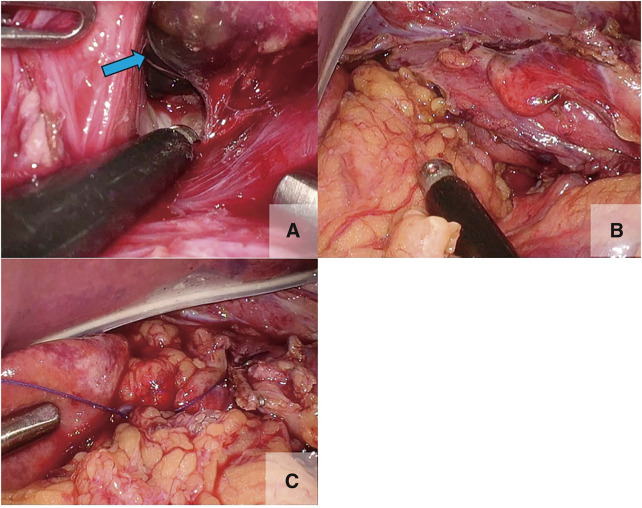
Intraoperative view. (**A**) The blue arrow shows the suspected site of partial necrosis of the esophageal wall. (**B**) The perforation site was filled with mobilized greater omentum. (**C**) The inserted greater omentum was fixed to the right curs of the diaphragm with an absorbable suture.

We performed laparoscopic surgery with five ports at a pneumoperitoneum pressure of 10 mmHg, according to laparoscopic gastrectomy. No peritoneal fluid contamination was observed. A portion of the dark red esophageal wall was noted upon retraction of the esophagus caudally using the tape. We incised the right crus of the diaphragm, opened the mediastinum and right thoracic cavity, and discharged an intermediate amount of dark-red fluid from the same area. The esophageal wall in this region had partially turned black (**Fig. 3A**), and partial necrosis was suspected. Given the difficulty of suturing the esophageal wall and the patient’s clinical condition, we assessed whether complete esophagectomy would also be challenging. Therefore, we mobilized the greater omentum from the transverse colon and filled the mediastinum to cover the site of the esophageal perforation (**Fig. 3B**). The inserted greater omentum was fixed to the right crus of the diaphragm by using an absorbable suture (**Fig. 3C**). The drains were positioned on the ventral side of the perforation site of the esophagus and the dorsal side of the esophagogastric junction. Before completing the surgery, a jejunostomy was performed, and a chest tube was inserted into the right thoracic cavity. The total operative time was 183 min and the total bleeding volume was 30 mL.

**Fig. 4:**
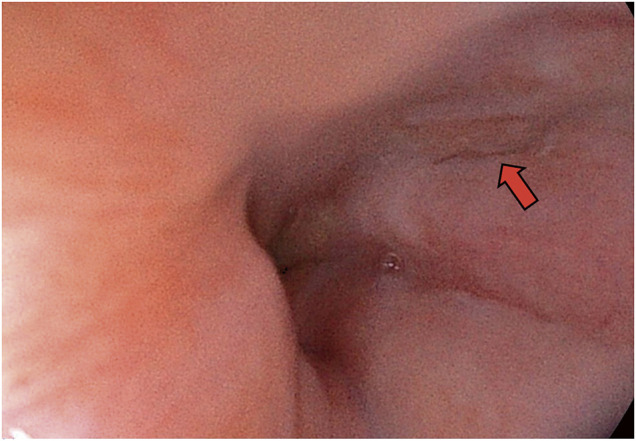
Endoscopic view (19 days after operation). The red arrow shows scar formation.

After surgery, the patient was admitted to the intensive care unit (ICU) for mechanical ventilation. Although vasopressors were initially administered, the dosage was gradually reduced, and enteral nutrition via jejunostomy was initiated on the 2nd postoperative day. The patient was extubated on the 4th postoperative day. The drain from the dorsal esophagogastric junction was removed on the 6th postoperative day. A contrast study of the gastric tube was performed on the 7th postoperative day, which confirmed the absence of significant leakage. On the 9th postoperative day, both the chest tube and the ventral esophageal drain were removed. Videofluorography was performed on the 14th postoperative day, and after confirmation of no leaks, the patient began drinking water. On the 19th postoperative day, upper gastrointestinal endoscopy revealed a scar in the lower esophagus (**Fig. 4**). The patient showed signs of healing, and oral intake was initiated on the 20th postoperative day. The patient had no problems with dietary intake and was discharged in a stable condition on the 24th postoperative day. Subsequent outpatient follow-ups revealed no symptom recurrence.

## DISCUSSION

Spontaneous esophageal rupture is a condition characterized by the perforation of all layers of the esophagus due to a rapid increase in intraluminal pressure, which is considered to occur as a result of an acute elevation in esophageal pressure due to vomiting.^1)^ The most commonly affected site was the left side of the lower esophagus, which is anatomically predisposed.^2)^ In this case, the onset was marked by sudden right chest pain, and although the perforation occurred in the lower esophagus, it was on the right side. Additionally, intraoperative findings suggested possible necrosis of the esophagus, indicating a potential secondary perforation; however, the lack of pathological findings left the precise cause undetermined.

Regarding the diagnosis of spontaneous esophageal rupture, CT has excellent sensitivity for detecting direct or indirect signs of esophageal perforation (extraluminal air bubbles or esophageal wall thickening).^3)^ In addition, esophagography or endoscopy is useful for confirming the perforation site. In this case, a gastric tube was placed in the esophagus for decompression, and contrast material was injected via the gastric tube to identify the perforation site.

Some reports have demonstrated the usefulness of conservative treatment for esophageal ruptures.^4,5)^ The indications for conservative management include: 1) contamination confined to the mediastinum, 2) adequate drainage into the esophagus through the rupture site, 3) mild clinical symptoms, 4) absence of severe infection, and 5) the patient’s overall condition remaining stable, allowing for more than 24 h of observation.^6,7)^ In this case, the symptoms were severe and the patient exhibited significant acidosis; therefore, we decided to perform emergency surgery.

No standard therapeutic strategies or specific surgical guidelines exist for the management of esophageal ruptures. Nonetheless, surgical repair of defects and drainage of the mediastinum has long been considered the gold standard for early perforations.^8)^ The fundamental surgical approach consists of early suturing and closure of the ruptured layer; thorough irrigation of the surrounding tissue, mediastinum, and thoracic cavity; and appropriate drainage. Primary closure is generally feasible within approximately 24 h post-onset.^9)^ However, owing to vascular insufficiency and a lack of serosa in the anatomical structure of the esophagus, even cases that undergo surgery relatively soon after onset have a high rate of dehiscence.^10)^ Consequently, various techniques have been developed to cover perforations using the omentum, stomach fundus, diaphragm, and pleura to reduce the incidence of dehiscence. Among these, the omentum is often regarded as ideal for coverage because of its mobility and properties that promote wound healing and adhesion.^11)^

In cases of extensive rupture with significant mediastinal contamination and challenging closure, thorough lavage is essential. If primary closure is not feasible, options such as thoracic esophagectomy, cervical esophageal fistula construction, and gastrostomy may be considered, followed by reconstructive surgery once the patient’s condition stabilizes.^12)^ Conventionally, thoracotomy is the preferred approach; however, thoracoscopic surgery is emerging as a standard and effective operative procedure for spontaneous esophageal rupture.^13)^ Furthermore, reports increasingly highlight the advantages of transabdominal approaches, particularly for improved omental coverage,^14)^ although laparoscopic interventions are limited. However, published case reports have reported the successful management of esophageal rupture via laparoscopy, with benefits that outweigh those of thoracoscopy or endoscopy.^15)^ Criteria for the appropriateness of laparoscopic surgery have been suggested, including the presence of localized abscesses surrounding the lower esophagus on preoperative imaging, low likelihood of thoracic penetration, and the surgeon’s proficiency in managing gastric cardia using laparoscopic techniques.^13)^ In this case, a small right pleural effusion was noted. However, given that the perforation was in the lower esophagus and the thoracic cavity was limited, we initially opted for laparoscopic surgery, with the possibility of converting to thoracotomy or laparotomy if needed. The perforation site appeared necrotic, making suturing difficult. However, we believe that adequately filling the mediastinum with omentum can effectively cover and seal the esophageal perforation. We achieved positive treatment outcomes by ensuring thorough drainage of both the perforation site and the right thoracic cavity. Even when the perforation extended into the thoracic cavity if contamination remained localized, laparoscopic drainage via the esophageal hiatus combined with chest drain placement effectively removed contaminants. We suggest that even when esophageal perforation closure is not feasible, laparoscopic omental filling can effectively cover and seal the perforation site to facilitate healing.

## CONCLUSIONS

Laparoscopic surgery is considered beneficial even for spontaneous esophageal rupture, particularly when suture closure of the perforation site is challenging. We present a case in which, despite the inability to suture the esophageal perforation, thorough lavage, drainage, and laparoscopic omental filling around the perforation site facilitated successful healing.

## DECLARATIONS

### Funding

The authors did not receive support from any organization for the submitted work.

### Authors’ contribution

DI drafted this manuscript.

HS was the main surgeon and reviewed and modified the manuscript.

KH and MT assisted during surgery.

All authors read and approved the final manuscript.

All authors agree to be responsible for all aspects of this study.

### Availability of data and materials

The datasets supporting the conclusions of this article are included within the article.

### Ethical approval and consent to participate

Not applicable.

### Consent for publication

Informed consent was obtained from the patient for publication of this case report.

### Competing interests

The authors declare that they have no competing interests.
